# Supplementary material for: Effects of β-1,6-Glucan Synthase Gene (FfGS6) Overexpression on Stress Response and Fruit Body Development in Flammulina filiformis
Source: Genes (Basel). 2022 Sep 28;13(10):1753. doi: 10.3390/genes13101753 (PMC9601887; doi:10.3390/genes13101753)
Supplement: Supplementary file 1 [file genes-13-01753-s001.zip › Supplementary material.pdf]

Table S1. Analysis of insertion sites of *FfGS6* overexpressing transformants.

Transformants	Sequence (black: genome sequence, line: vector sequence)	The position of plasmid	The position of genome
OE#10	>A00917:623:H75VHDSX2:4:1601:26476:9236 1:N:0:TCATGAGG+CTAGCACT<br>CACGAGGTCCAAGGTCCCGTCCAACCGTT<br>CGCTCAAACACCTCCATCATGAAGGTGAA<br>CATGGAACGCGTCAAGGCAGCTCCCTCGT<br>GCGCTCTCCTGTTCCGACCCTGCCGCTTAC<br>CGGATACCTGTCCGCCTTTCTCCCTTCGGG<br>AAG	3796-3869 bp	Fv01-10<br>Scaffold2: 1814110-1814185 bp
	>A00917:623:H75VHDSX2:4:1306:16703:1955 1:N:0:TCATGAGG+CTAGCACT<br>CCGCCGACCTGGTGGAGCTGGTTAAGCAG<br>CGCATTGAGGTCACGGATGGAAGGCTACA<br>AGCGGCCTTTGTCGAGGCCGTCGGCGTCG<br>TTCACGACGGCGTCCAGAGCACGAGAAA<br>GGCAGCGAGAAGTGGTGGTGGTTAAGTGA<br>TGACT	972-1043 bp	Fv01-10<br>Scaffold2: 1813599-1813675 bp
OE#12	>A00917:623:H75VHDSX2:4:2376:2926:35243 1:N:0:CCTGTAGT+GAGTCAAC<br>CAGCAAACGTGCCCATCCAGGAGACGTAC<br>CAAGCACTGGAACAGCTTGTGGACGATGG<br>TCTTGCCAAGAACATTGGTGTTAGCAACA<br>GGATATATTGTGGTGTAAACAAATTGACG<br>CTTAGACAACTTAATAACACATTGCGGAC<br>GTTTT	5587-5651 bp	Fv01-10<br>Scaffold2: 2800030-2800114 bp
	>A00917:623:H75VHDSX2:4:1429:2790:14465 1:N:0:CCTGTAGT+GAGTCAAC<br>ATGCCGAATGCTAGAGCAGCTTGAGCTTG<br>GATCAGATTGTCGTTTCCCGCCTTCAGTTT<br>AAACTATCAGTGTTTGAATGTATTTCATCA<br>TTGGCGTCCTTCGCTATGCTCGGTACCCAC<br>CCCAGGTGCTGCAAGTAGAGCTGCACCCG<br>TA	10219-10295 bp	Fv01-10<br>Scaffold2: 2799941-2800014 bp
OE#14	>A00917:623:H75VHDSX2:4:1559:1072:16219 1:N:0:CGTCTTAG+TAGCACAG<br>CGTACTGAGCGTCTCGATTGTTGGCCGCCA<br>GACTTCTTGCTTAATTGCTGATTTCACGTG<br>CTCGTGGTGTAAACAAATTGACGCTTAGA<br>CAACTTAATAACACATTGCGGACGTTTTTA<br>ATGTACTGAATTAACGCCGAATTAATTCG<br>GG	5600-5684 bp	Fv01-10<br>Scaffold4: 2822231-2822295 bp
	>A00917:623:H75VHDSX2:4:1229:28800:32268 1:N:0:CGTCTTAG+TAGCACAG<br>AACAGTTGCGCAGCCTGAATGGCGAATGC<br>TAGAGCAGCTTGAGCTTGGATCAGATTGT<br>CGTTTCCCGCCTTCAGTTTAAACTATCAGT<br>GTTTGATTATTGTGGAATGAAGTCTCTTGC<br>CATCTCTTCACTCCTTACCACCTTCTCCAA<br>CC	10202-10295 bp	Fv01-10<br>Scaffold4: 2822332-2822387 bp

OE#16	>A01415:29:H3TGJDSX2:2:2459:21278:12289 1:N:0:AGACCTGTTC+AGGCTAACAC<br>ATCGTTGAGGAAAGGAGGAAAGGAGGAT<br>CCAAACTAACAACTGACCCGTTTCCGTAA<br>CGACGACAGGATATATTGTGGTGTAAACA<br>AATTGACGCTTAGACAACTTAATAACACA<br>TTGCGGACGTTTTTAATGTACTGAATTAAC<br>GCCGA	5587-5673 bp	Fv01-10<br>Scaffold13: 1363645-1363707 bp
	>A01415:29:H3TGJDSX2:2:1535:3821:36479 1:N:0:AGACCTGTTC+AGGCTAACAC<br>TAGAGCAGCTTGAGCTTGGATCAGATTGT<br>CGTTTCCCGCCTTCAGTTTAAACTATCAGT<br>GCGTCCGCCGGCCCTGAACTATTATAACG<br>ATAATTGATCAAGAATTCCCCGGCACTTTT<br>ACGTCCACTTGCATCTTGCCTCCATCTCAT<br>CT	10231-10290 bp	Fv01-10<br>Scaffold13: 1368533-1368622 bp

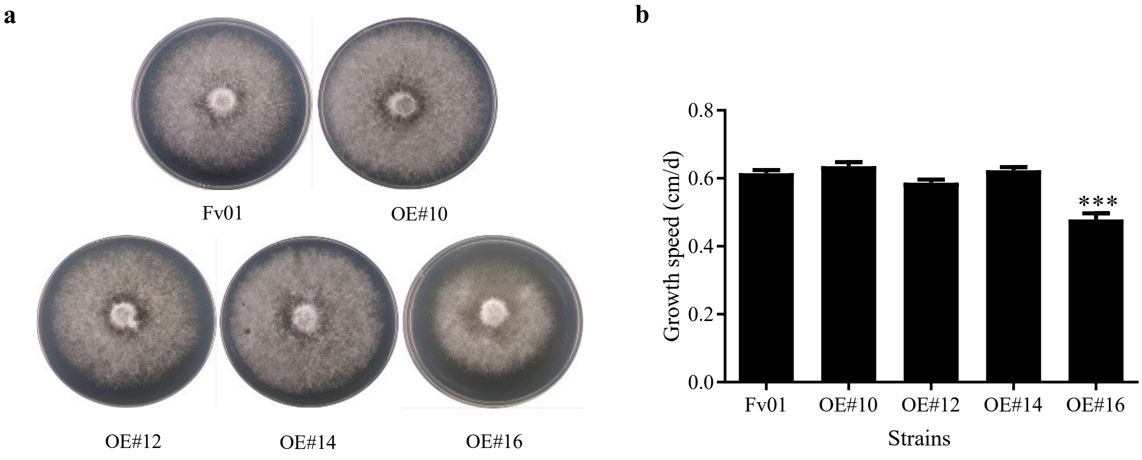

Figure S1. Mycelial phenotype analysis of *Flammulina filiformis FfGS6* overexpression transformants. (a) The mycelial phenotype on PDA plates; (b) The mycelial growth rate on PDA plates. Asterisks represent significant differences versus Fv01 (*t*-test, *n* = 9; *, *p* < 0.001).**

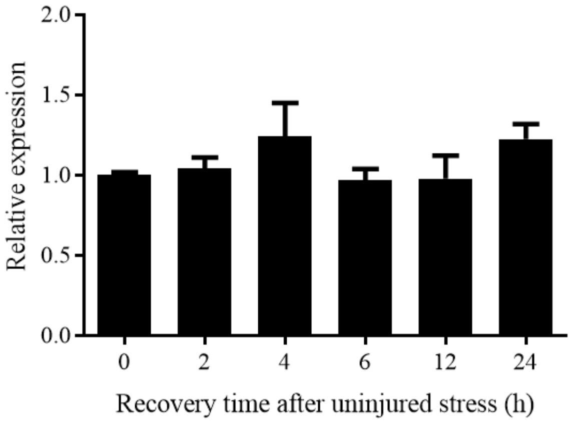

Figure S2. *FfGS6* gene expression at different recovery time points (0, 2, 4, 6, 12, and 24 h, respectively) after Fv01 mycelial uninjured induction. (*t*-test, *n* = 3).
